# Transcription Factor SOX10 Improves Migration and Homing of MSCs After Myocardial Infarction by Upregulating CXCR4

**DOI:** 10.1155/sci/1880402

**Published:** 2025-05-26

**Authors:** Baoping Deng, Qili Liu, Jiemin Yang, Jing Xu, Hongmei Zheng, Weiping Deng

**Affiliations:** ^1^Department of Vascular Surgery, Affiliated Hospital of Guilin Medical University, Guilin 541001, China; ^2^Department of Interventional Vascular Surgery, The Fifth Affiliated Hospital, Southern Medical University, 566# Congcheng Road, Conghua District, Guangzhou, Guangdong Province, China; ^3^Department of Gastroenterology, Shenzhen Longhua District Central Hospital, 187# Guan Lan Road, Longhua District, Shenzhen 518110, Guangdong, China

**Keywords:** CXCR4, homing, MI, MSCs, SOX10

## Abstract

**Background:** CXCR4 enhances the homing of mesenchymal stem cells (MSCs), thereby potentially improving outcomes in myocardial infarction (MI). However, the molecular mechanisms underlying MSC homing remain poorly understood.

**Methods:** The identity of MSCs was confirmed through flow cytometry, utilizing their cluster of differentiation (CD) marker profile. Migration and invasion were assessed using wound healing and transwell assays. In a rat MI model, myocardial function, hemodynamic parameters, and the degree of myocardial fiber damage were evaluated post-MSC treatment, along with the observation of MSC homing. Luciferase assays identified binding sites between SOX10 and the CXCR4 promoter, and the effects of SOX10 on MSC migration, invasion, and homing were explored both *in vitro* and *in vivo*.

**Results:** Overexpression of CXCR4 significantly enhanced MSC migration, invasion, and homing. MSCs overexpressing CXCR4 improved cardiac function and reduced infarct size in the rat MI model. A direct interaction between SOX10 and CXCR4 was confirmed, with SOX10 acting as a transcription factor to upregulate CXCR4 expression, thereby enhancing MSC homing and ameliorating MI in rats. Knockdown of SOX10 reversed the beneficial effects of CXCR4-overexpressing MSCs on MI therapy, as well as the functional impact of CXCR4 on MSCs.

**Conclusion:** In conclusion, SOX10 facilitates MSC homing by upregulating CXCR4 expression, offering a potential therapeutic approach for MI treatment.

## 1. Introduction

Myocardial infarction (MI) is a potentially fatal cardiovascular condition that leads to ischemia, necrosis, and fibrosis within the heart. MI is one of the leading global causes of death, responsible for 17% of all fatalities annually [1]. Despite advances in medical technologies and reperfusion therapies, including direct arterial perfusion and thrombolytic treatments, which improve clinical outcomes and save lives, these interventions fail to fundamentally reverse or halt the progression of heart failure post-MI [2, 3]. As a result, novel therapeutic strategies are urgently needed to mitigate post-MI remodeling and prevent heart failure.

Stem cell therapy has emerged as a promising treatment modality capable of providing functional cells to regenerate damaged cardiac tissue [4]. However, effective stem cell migration and homing to the injured cardiac tissue remain significant challenges.

Mesenchymal stem cells (MSCs) are adult stem cells with self-renewal and multipotent differentiation capabilities. They are easy to isolate and expand *in vitro* [5] and are increasingly utilized in clinical applications, including MI treatment [6].

MSC homing refers to the process by which these cells migrate to and engraft within specific tissues under various stimuli [7]. While MSCs possess inherent homing properties, their ability to migrate and home to cardiac tissue is limited, hindering their therapeutic efficacy in MI treatment [8]. Current challenges in MSC transplantation include ensuring a sufficient number of cells successfully home to the pathological tissue, as low homing efficiency compromises both hematopoietic regeneration and tissue repair. Conversely, enhancing MSC migration to target organs has been shown to improve clinical outcomes significantly [9].

Enhancing the migration of MSCs to target organs could improve the success rates of MSC transplantation, as effective *in vivo* migration is essential for their therapeutic function [10]. CXCR4 (C-X-C chemokine receptor type 4) is a G protein-coupled receptor widely expressed on various cell types, including hematopoietic, endothelial, epithelial, neuronal, progenitor, and stem cells [11]. CXCR4 binds to stromal-derived factor-1*α* (SDF-1*α*), which directs stem cells, particularly MSCs, to damaged tissues such as those affected by MI [11–13]. The SDF-1/CXCR4 axis plays a pivotal role in MSC migration and homing *in vivo* [11, 13].

This study aims to evaluate the migration and homing potential of MSCs through *in vitro* and *in vivo* experiments, investigate the mechanism of CXCR4 action, and explore the regulatory role of CXCR4 in MSC migration. The findings from this study are expected to provide a theoretical foundation for enhancing the effectiveness of MSC-based therapies in MI treatment and contribute to the advancement of cardiac regenerative medicine.

## 2. Methods

### 2.1. Isolation and Culture of Human MSCs

Bone marrow was harvested from the posterior iliac crest of healthy adult volunteers undergoing orthopedic surgery, following the procedure described previously [14]. Nucleated cells were isolated using a density gradient (Lymphoprep, Stemcell Technologies, Inc., Vancouver, Canada). A total of 1.2 × 10^7^ nucleated cells were plated in Dulbecco's Modified Eagle's Medium (DMEM; GIBCO, Grand Island, NY) and incubated at 37°C in a humidified incubator with 5% CO_2_. After 24 h, nonadherent cells were discarded, and adherent cells were washed twice with PBS. The culture medium was replaced every 3 days. Once the cells reached 80% confluence after 10 days, they were treated with 0.05% trypsin-EDTA (Gibco) for 5 min at 37°C and then replated at a density of 2500 cells/cm^2^ in T75 tissue culture flasks. After 5 additional days, the cultured cells were either harvested or expanded for further experiments.

### 2.2. Flow Cytometry

The cells were harvested, washed, and resuspended. The antibodies included anti-CD73-FITC (BD Biosciences, Franklin Lakes, NJ, USA, 1:2000), anti-CD90-FITC (BD Biosciences, 1:2000), anti-CD105-FITC (BD Biosciences, 1:3000), anti-CD11b-PE (BD Biosciences, 1:2000), anti-CD14-FITC (BD Biosciences, 1:3000), anti-CD34- PE (BD Biosciences, 1:2000), and anti-CD45-FITC (BD Biosciences, 1:5000) were added to the cells at 4°C for 1 h. Furthermore, cells were analyzed on a BD FACSCalibur (BD Biosciences).

### 2.3. Oil Red O Staining

To assess adipogenesis, terminal adipogenic cultures were stained with Oil Red O to detect lipid droplet accumulation, a key feature of adipocytes [15]. MSCs were fixed in 4% paraformaldehyde (PFA) at room temperature for 10 min, followed by fixation of the coverslips in formaldehyde-calcium for 10 min at 37°C. After washing with distilled water, the samples were rinsed with 60% isopropanol and stained with Oil Red O (Beyotime Institute of Biotechnology) at 37°C for 10 min. The staining was differentiated using 60% isopropanol, followed by washing with distilled water. Hematoxylin counterstaining was performed at 37°C for 10 min, and the samples were rinsed with water before microscopic examination under a light microscope. Image J software was used to quantify the contents of lipids by calculating the area of red lipid droplets.

### 2.4. Lentiviruses and Infection

Overexpression and knockdown of CXCR4 and SOX10 in MSCs were performed through the transfection of lentiviruses (GenePharma, Shanghai, China). MSCs were plated in T25 culture flasks and incubated overnight, followed by a wash with PBS. Subsequently, 2 mL of culture medium devoid of penicillin/streptomycin was added to each flask. Lentivirus was then introduced directly into the culture medium (MOI = 20). After 16 h, the lentivirus-containing medium was replaced with a fresh complete medium. Following a 72-h incubation period, puromycin (Sigma–Aldrich, St. Louis, MO, USA) was added to the medium for 1 week to screen stably infected MSCs. Ultimately, the transfection efficiency was assessed using RT-qPCR and Western blotting. The sequences of shRNA used in the study were as follows: sh-CXCR4: Forward: 5′-ACCGCGATCAGTGTGAGTATATAAAGT-3′, Reverse: 5′-TCTCTTATATACTCACACTGATCGCTTTTTC-3′; sh-SOX10: Forward: 5′-CTGCTGTTCCTTCTTGACCTTGCCC-3′, Reverse: 5′-TCCTTCTTCAGATCGGGCT-3′.

### 2.5. RT-qPCR

Total RNA was extracted from the cells using TRIzol reagent (Invitrogen, Waltham, Massachusetts). Reverse transcription of total RNA was performed using the PrimerScript Real-time reagent kit (TaKaRa, Kusatsu, Shiga, Japan). Quantitative analysis of CXCR4 and SOX10 expression was carried out using SYBR Premix Ex TaqTM II (TaKaRa, Japan). The primer sequences used for real-time PCR were CXCR4: Forward: 5′-ACAGGTACATCTGTGACCGCCTTT-3′, Reverse: 5′-GATGAAGGCCAGGATGAGAA-3′; SOX10: Forward: 5′-TGCTCTCGAAGTCACATCCTTGCT-3′, Reverse: 5′-CATATAGGAGAAGGCCGAGTAGA-3′; GAPDH: Forward: 5′-ACCACAGTCCATGCCATCAC-3′, Reverse: 5′-TCCACCACCCTGTTGCTGTA-3′.

### 2.6. Western Blotting

Protein lysates were prepared from cells using IP lysis buffer containing protease and phosphatase inhibitors (Pierce, Thermo Fisher Scientific). The total protein concentration was quantified using the BCA Protein Assay Kit (Pierce, Thermo Fisher Scientific). Equal amounts of protein were separated by sodium dodecyl sulfate-polyacrylamide gel electrophoresis (SDS-PAGE) and transferred onto a PVDF membrane (Millipore, Bedford, MA, USA). The membrane was blocked with 5% non-fat milk at room temperature for 1 h, followed by overnight incubation with primary antibodies at 4°C. Primary antibodies used were CXCR4 (1:2000; Abcam, Cambridge, UK), SOX10 (1:2000; Abcam, Cambridge, UK), and GAPDH (1:2000; Abcam, Cambridge, UK). After incubation with appropriate secondary antibodies for 1 h at room temperature, protein detection was carried out using ECL reagents (Beyotime, Haimen, China).

### 2.7. Wound Healing Assay

For the migration assay, cells were seeded in six-well plates and allowed to reach confluency. A sterile pipette tip was used to create a scratch wound on the cell monolayer. Cells were then incubated in a serum-free medium for 24 h to induce migration. Wound closure was monitored by capturing images with an inverted microscope and digital camera at 0 and 24 h postscratch. The distance traveled by the cells was measured using ImageJ software.

### 2.8. Transwell Assay

The treated cells were seeded in the upper chamber of a 24-well Transwell system at a density of 2 × 10^5^ cells per well. The upper chamber was coated with Matrigel, and 600 μL of culture medium containing 10% FBS was added to the lower chamber. After 24 h of incubation, the transwell chamber was carefully removed, and cells were fixed with 4% PFA for 20 min. The chamber was then washed with PBS, and cells on the chamber surface were wiped off using cotton swabs. After crystal violet staining, the cells were observed under a microscope. Six random visual fields were selected for imaging and cell counting. The experiment was independently repeated three times.

### 2.9. Establishment of MI Rat Models and Identification

Forty healthy male SD rats (250 ± 10 g) were obtained from the Animal Experimental Center. All animal procedures were approved by the Ethics Committee of Shenzhen Longhua District Central Hospital and conducted in compliance with the U.S. Public Health Service Guide for the Care and Use of Laboratory Animals, with efforts to minimize animal suffering and reduce animal usage. Rats were anesthetized with an intraperitoneal injection of 2% pentobarbital sodium (40 mg/kg; Sigma–Aldrich) and positioned on the operating table. The MI model was induced by ligating the left anterior descending branch of the coronary artery [16].

### 2.10. Animal Grouping

7 days post-MI model establishment, rats were transplanted with BMSCs and divided into five treatment groups (*n* = 5/group): (i) Sham group, rats subjected to thoracotomy without coronary artery ligation; (ii) MI group, untreated MI rats; (iii) MSC group, MI rats receiving MSC injections *via* the caudal vein; (iv) MSC-CXCR4 group, MSCs overexpressing CXCR4; (v) MSC-NC group, MSC transplantation with negative control.

In subsequent experiments, rats were divided into three groups (*n* = 5/group) based on different treatments: (i) MSC-ctrl group, negative control; (ii) MSC-SOX10 group, MSCs overexpressing SOX10; (iii) MSC-SOX10 + sh-CXCR4 group, MSCs overexpressing SOX10 with CXCR4 knockdown.

### 2.11. Cardiac Function Assessment

Four weeks post-transplantation of MSCs, echocardiography was performed using a Vevo 2100 system (VisualSonics, Toronto, ON, Canada) equipped with a 21-MHz transducer. Rats were anesthetized with 2% pentobarbital sodium (40 mg/kg; Sigma–Aldrich) during the procedure. LV end-diastolic and end-systolic dimensions (LVEDD, LVESD) were measured from the parasternal short-axis view at the level of the papillary muscle using M-mode tracing [17]. LV ejection fraction (LVEF) was calculated using the following formula: LVEF (%) = (LVEDD^3^ - LVESD^3^)/LVEDD^3^ × 100. LV fractional shortening (LVFS) was calculated as follows: LVFS (%) = (LVEDD - LVESD)/LVEDD × 100.

### 2.12. Masson's Staining

Tissue sections were stained with Weigert's iron hematoxylin working solution for 10 min, followed by differentiation in acid ethanol solution for 15 s, and immersion in Masson's blue solution for 4 min. Subsequently, sections were stained with carmine fuchsin for 6 min and water blue solution for 2 min. Staining results were observed under a light microscope (OLYMPUS).

### 2.13. Database Selection and Analysis

In this study: JASPAR (http://www.jaspar.genereg.net) was used for the prediction of potential binding sites of SOX10 and CXCR4 promoters.

### 2.14. Dual-Luciferase Reporter Assay

The CXCR4 promoter region, containing a putative SOX10 binding site, was PCR-amplified and inserted into the psiCHECK-2 luciferase reporter plasmid. The luciferase reporter plasmids were transfected into SOX10-overexpressing MSCs. Luciferase activity was measured 48 h post-transfection using a dual-luciferase reporter assay kit (Promega). Data were expressed as the Renilla/firefly luciferase ratio.

### 2.15. Chromatin Immunoprecipitation Assay

Chromatin immunoprecipitation (ChIP) was conducted using a ChIP assay kit (Beyotime) following the manufacturer's instructions. Briefly, MSCs were cross-linked with formaldehyde and then sonicated to achieve an average fragment size of 200–500 base pairs. The cell lysates were precleared using protein A/G beads, followed by incubation with protein A/G beads coated with the anti-SOX10 antibody (Abcam). Anti-rabbit IgG served as a negative control. The cross-linked DNA released from the protein–DNA complex was purified using a DNA Extraction Kit (GeneMark), and the eluted DNA was subsequently analyzed using RT-qPCR. The specific primers utilized for ChIP-qPCR are as follows: CXCR4: Forward: 5′- GGTAGGGTGCAGCTTACGGTC-3′, Reverse: 5′- ACGGTAGATCCTATCTACTGAAGG-3′.

### 2.16. Statistical Analysis

Statistical analysis was performed using GraphPad Prism 7 software. Comparisons between two groups were made using the Student's *t*-test. Differences among multiple groups were analyzed using ordinary one-way ANOVA with Tukey's multiple comparisons test. Data are presented as means ± standard deviation (SD).

## 3. Results

### 3.1. Isolation and Identification of Human MSCs

**Figure 1:**
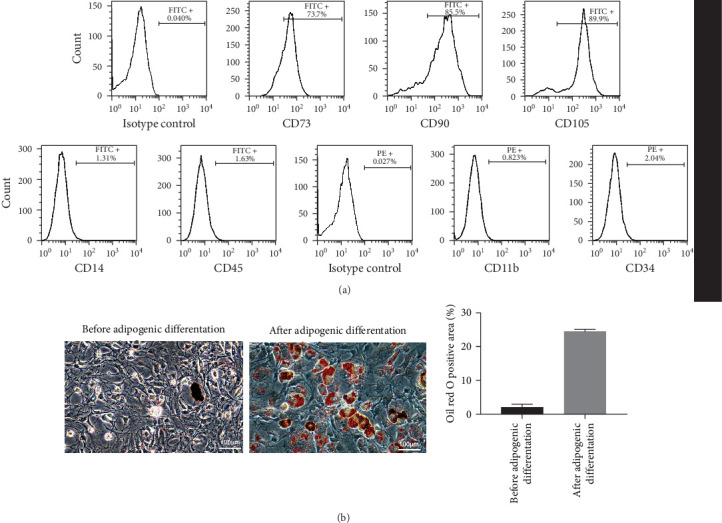
Isolation and culture of human mesenchymal stem cells (MSCs). (a) Flow cytometry analysis of MSC surface markers. (b) ORO staining of MSCs (magnification, ×200).

The phenotypic characteristics of MSCs were analyzed using flow cytometry. The results showed that CD73, CD90, and CD105 were positively expressed on the surface of MSCs, while CD11b, CD14, CD34, and CD45 were negative (Figure 1a). MSCs cultured under adipogenic-inductive conditions exhibited adipogenic differentiation potential, as indicated by the presence of lipid droplets (Figure 1b). These results confirm successful MSC culture.

### 3.2. CXCR4 Promoted the Invasion and Migration of MSCs

**Figure 2:**
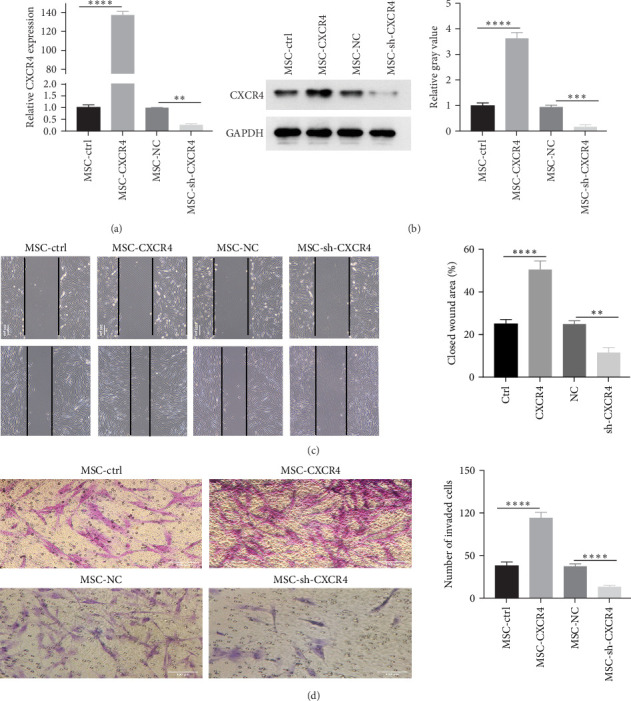
CXCR4 promotes invasion and migration of MSCs. (a) RT-qPCR detection of CXCR4 expression in MSCs transfected with interference and overexpression lentiviruses. (b) Western blot analysis of CXCR4 expression in MSCs. (c) Wound healing assay to assess MSC migration ability. (d) Transwell assays to measure MSC invasion ability. MSC, mesenchymal stem cell. *⁣*^*∗∗*^*p* < 0.01, *⁣*^*∗∗∗*^*p* < 0.001, and *⁣*^*∗∗∗∗*^*p* < 0.0001.

To explore the biological role of CXCR4 in MSCs, CXCR4 overexpression or knockdown lentiviruses were infected into MSCs. As shown in Figure 2a,b, and Supporting Information 1: Figure S3, the CXCR4 overexpression or knockdown efficiency was verified using RT-qPCR and Western blotting. Additionally, migration of MSCs was increased after CXCR4 overexpression and decreased after CXCR4 downregulation (Figure 2c). Moreover, the transwell assay was performed to elucidate the potential role of CXCR4 in regulating the invasion of MSCs. CXCR4 overexpression significantly promoted the invasion of MSCs, whereas CXCR4 knockdown had the opposite effect (Figure 2d). These results indicated that CXCR4 overexpression could promote the migration and invasion of MSCs.

### 3.3. MSCs-Overexpressing CXCR4 Improved Cardiac Functions in a Rat MI Model

**Figure 3:**
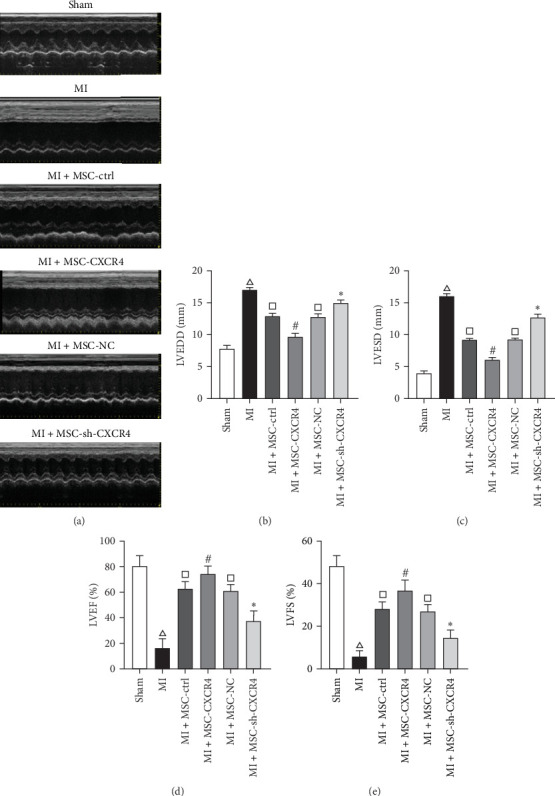
MSCs overexpressing CXCR4 improve cardiac function in a rat MI model. (a) Echocardiography assessing cardiac function 4 weeks after MI induction and MSC transplantation. (b) Quantification of LVEDD. (c) Quantification of LVESD. (d) Quantification of LVEF. (e) Quantification of LVFS. *⁣*^*∗*^*p*  < 0.05 compared to the NC group, ^#^*p*  < 0.05 compared to the Ctrl group, ^△^*p*  < 0.05 compared to the Sham group, ^□^*p*  < 0.05 compared to the MI group. MSC, mesenchymal stem cell.

Echocardiography was performed 4 weeks after MSC implantation in a rat MI model (Supporting Information 2: Figure S1) to assess the therapeutic effects of MSCs on cardiac function (Figure 3a). LVEDD and LVESD were significantly reduced in the MSC-CXCR4, MSC-ctrl, and MSC-NC groups, while they were significantly higher in the MI and MSC-sh-CXCR4 groups (Figure 3b,c). Furthermore, the MSC-CXCR4, MSC-ctrl, and MSC-NC groups exhibited significantly increased LVEF and LVFS, whereas the MI and MSC-sh-CXCR4 groups showed significantly reduced LVEF and LVFS (Figure 3d,e). These results collectively suggest that MSCs overexpressing CXCR4 can improve cardiac function.

### 3.4. CXCR4 Promoted the Homing of MSCs to Alleviate MI in Rats

**Figure 4:**
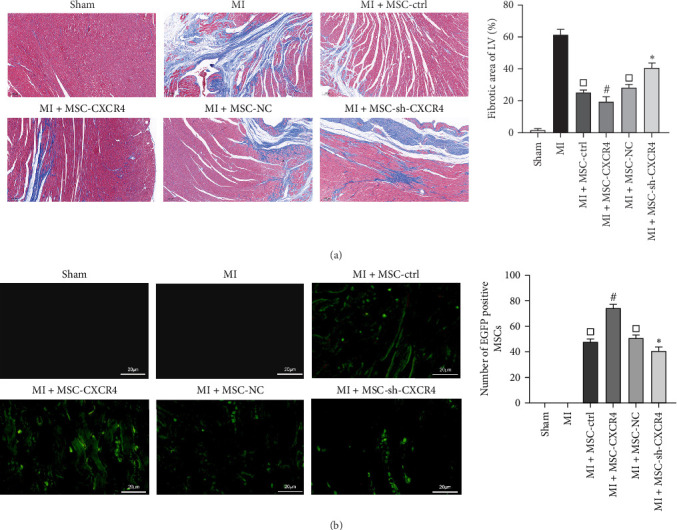
CXCR4 promotes MSC homing to alleviate MI in ratsa. (a) Masson staining of myocardial fibers in each group (*n* = 5/group) (magnification, ×200). (b) Fluorescence microscopy (magnification, ×400) to measure MSC homing ability in rat myocardium (*n* = 5/group). *⁣*^*∗*^*p*  < 0.05 compared to the NC group, ^#^*p*  < 0.05 compared to the Ctrl group, ^□^*p*  < 0.05 compared to the MI group. MI, myocardial infarction; MSC, mesenchymal stem cell.

To further investigate the role of MSC-CXCR4 in MI, Masson's trichrome staining was used to assess the extent of fibrosis after MSC treatment. The infarct size was significantly smaller in all MSC-treated groups compared to the MI group. Notably, the infarct size was smaller in the MSC-CXCR4 group compared to the MSC-ctrl group, whereas the MSC-sh-CXCR4 group exhibited a significantly larger infarct size compared to the MSC-NC group (Figure 4a). MSCs labeled with lentivirus-mediated green fluorescent protein were injected into the tail veins of MI rats. A greater number of MSCs were detected in the myocardium of the MI + MSC-CXCR4 group compared to the MI + MSC-ctrl group, indicating that MSCs overexpressing CXCR4 have enhanced homing efficiency. Conversely, the MI + MSC-sh-CXCR4 group showed reduced homing efficiency to the myocardium compared to the MI + MSC-NC group (Figure 4b). These results suggest that CXCR4 improves MI outcomes by enhancing MSC homing to the infarcted myocardium.

### 3.5. Knockdown of CXCR4 Reversed the Effects of SOX10 on the Migration and Invasion of MSCs

**Figure 5:**
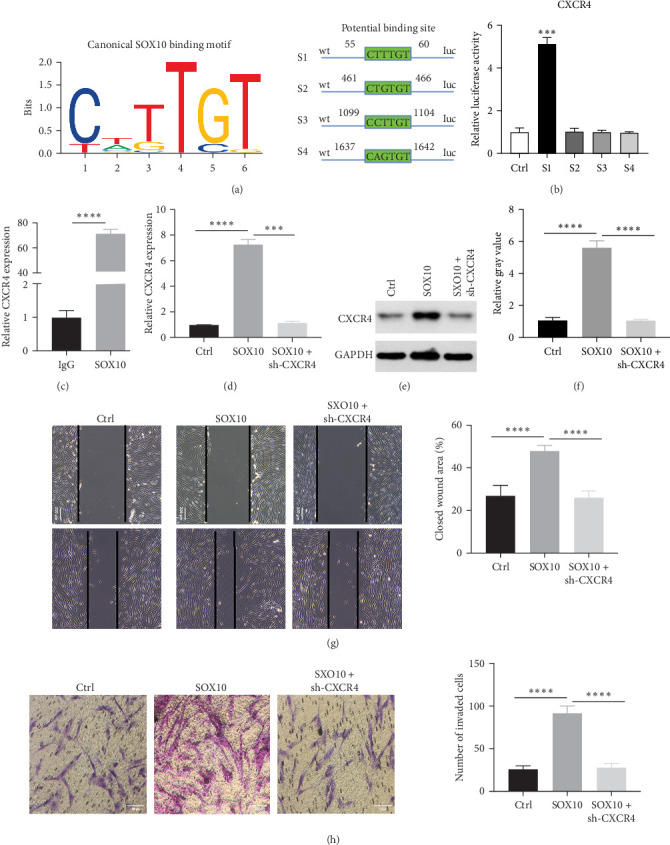
Knockdown of CXCR4 reverses SOX10-induced effects on MSC migration and invasion. (a) JASPAR database predicts binding sites between SOX10 and CXCR4. (b, c) Dual-luciferase reporter and ChIP assays were performed to detect the SOX10 binding to the CXCR4 promoter in MSCs. (d–f) The expression of CXCR4 by RT-qPCR and Western blotting. (g) Wound healing detected the cell migration ability. (h) Transwell assays detected the cell invasion ability. MSC, mesenchymal stem cell. *⁣*^*∗∗∗*^*p* < 0.001 and *⁣*^*∗∗∗∗*^*p* < 0.0001.

The JASPAR database was utilized to predict the potential binding sites (S1–S5) of SOX10 and CXCR4 promoters (Figure 5a). Luciferase assays were then conducted to assess the transcriptional activity of these potential binding sites in MSCs, revealing that SOX10 can bind to the CXCR4 promoter (Figure 5b). These results were further validated through ChIP assays (Figure 5c). Additionally, overexpression of SOX10 led to increased CXCR4 expression, which was reversed upon CXCR4 knockdown (Figure 5d–f, Supporting Information 1: Figure S3 and Supporting Information 3: Figure S2a,b). These results suggest that SOX10 may regulate MSC migration and invasion by modulating CXCR4 expression. Increased migration and invasion were observed in MSCs overexpressing SOX10, while CXCR4 knockdown reversed the promoting effects of SOX10 on MSC proliferation and invasion (Figure 5g,h). These results indicate that CXCR4 knockdown can reverse the migration and invasion-promoting effects of SOX10 in MSCs.

### 3.6. Knockdown of CXCR4 Inverted the Improvement Effect of SOX10-Overexpressing MSCs on Rats With MI

**Figure 6:**
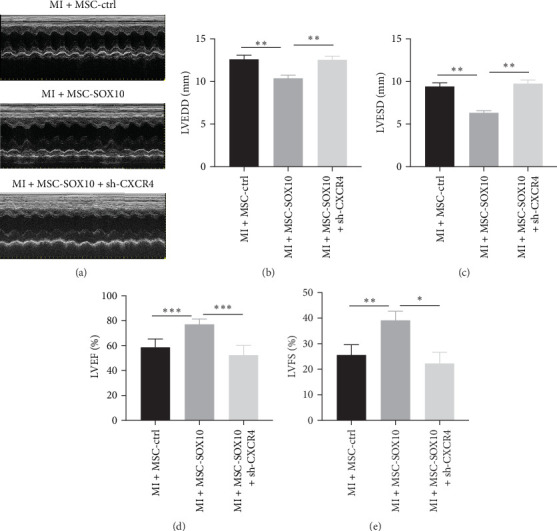
Knockdown of CXCR4 reverses the beneficial effects of SOX10-overexpressing MSCs in a rat MI model. (a) Echocardiography determining cardiac function. (b) Quantification of LVEDD. (c) Quantification of LVESD. (d) Quantification of LVEF. (e) Quantification of LVFS. LVEDD, LV end-diastolic dimensions; LVEF, LV ejection fraction; LVFS, LV fractional shortening; MI, myocardial infarction; MSC, mesenchymal stem cell. *⁣*^*∗*^*p* < 0.05, *⁣*^*∗∗*^*p* < 0.01, and *⁣*^*∗∗∗*^*p* < 0.0001.

To evaluate the impact of SOX10-overexpressing MSCs on cardiac function in a rat MI model, echocardiography was performed (Figure 6a, and Supporting Information 3: Figure S2c–g). LVEDD and LVESD were significantly reduced in the MSC-SOX10 group compared to the MSC-ctrl group (Figure 6b,c), but this effect was reversed in the MSC-SOX10 + sh-CXCR4 group. Additionally, the MSC-SOX10 group exhibited significantly increased LVEF and LVFS, whereas the MSC-SOX10 + sh-CXCR4 group showed significantly reduced LVEF and LVFS (Figure 6d,e). These results collectively suggest that SOX10-overexpressing MSCs enhance cardiac function, but this improvement is reversed by CXCR4 knockdown.

### 3.7. CXCR4 Knockdown Abolished the Promoting Effect of SOX10 on MSCs Homing

**Figure 7:**
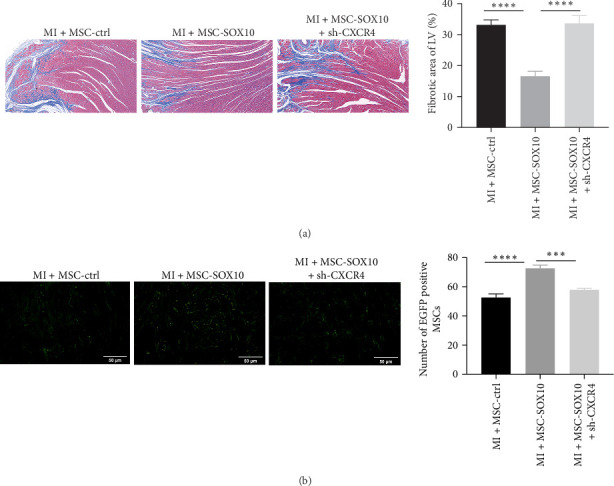
CXCR4 knockdown reverses MSC homing ability. (a) Masson staining of myocardial fibers in each group (*n* = 5/group) (magnification, ×200). (b) Fluorescence microscopy (magnification, ×400) to assess MSC homing in rat myocardium (*n* = 5/group). MSC, mesenchymal stem cell. *⁣*^*∗∗∗*^*p* < 0.001 and *⁣*^*∗∗∗∗*^*p* < 0.0001.

To further investigate the therapeutic mechanisms underlying SOX10-mediated cardiac repair, the infarct size and MSC homing in myocardial tissue were measured. The infarct size was significantly smaller in the MSC-SOX10 group compared to the MSC-ctrl group, while CXCR4 knockdown enlarged the infarct size (Figure 7a). Additionally, overexpression of SOX10 increased the number of MSCs homing to the myocardial tissue, whereas CXCR4 knockdown significantly reversed this effect (Figure 7b). These findings demonstrate that SOX10 improves MI outcomes by promoting MSC homing and that CXCR4 knockdown abolishes this effect.

## 4. Discussion

MI is a prevalent cardiovascular disease characterized by the permanent loss of cardiomyocytes and the subsequent formation of scar tissue due to myocardial ischemia [18]. MSCs are currently employed in MI treatment, with their therapeutic mechanisms including homing to and repairing damaged tissues [19, 20]. Despite their inherent homing ability, MSCs exhibit low migration efficiency to injured tissues *in vivo*, and the success of transplantation is closely tied to the number of cells that successfully home to the target site [21]. Therefore, understanding the mechanisms behind MSC homing and developing strategies to enhance their recruitment is crucial for achieving optimal transplantation outcomes.

CXCR4 is present on the surface of different types of stem cells. Recent research has revealed that CXCR4 is critical for the migration and homing of stem cells [22]. CXCR4 overexpression significantly enhances the migratory capacity of BMSCs toward inflammation sites [23]. Additionally, the CXCR4 antagonist AMD3100 facilitates tissue regeneration by modulating the homing of MSCs [24]. Furthermore, silencing CXCR4 expression inhibited the migration and homing of BM-MSCs to the damaged lung tissue [22]. Moreover, ultrasound-targeted microbubble destruction upregulated the expression of surface CXCR4 on MSCs, and CXCR4-positive MSCs can migrate and home to the ischemic myocardium [25]. Consistent with the above findings, the present study found that CXCR4 overexpression promoted the migration and invasion of MSCs. Furthermore, CXCR4 increased the migration and homing ability of MSCS to ameliorate MI, thereby enhancing cardiac function.

JASPAR predicts that SOX10 can target CXCR4. Chip and dual-luciferase reporter assays validated that SOX10 directly bound to the CXCR4 promoter. Additionally, overexpression of SOX10 increased CXCR4 expression. SOX10 belongs to the SOX family and is involved in regulating stem cell states, directing differentiation, and the control of stem/progenitor activity and epithelial–mesenchymal transition (EMT) [26, 27]. SOX10 is specifically expressed in mammary cells exhibiting the highest levels of stem/progenitor activity, and the deletion of SOX10 reduced stem/progenitor functions, whereas its ectopic activation not only enhanced stem/progenitor activity but also triggered EMT [28]. Moreover, SOX10 has been implicated in various biological processes, such as blood vessel tissue remodeling, proliferation, and differentiation, as observed in both regenerating tissues and tumors [27]. SOX10 + adult stem cells contribute to biomaterial encapsulation and microvascularization [29]. In nasopharyngeal carcinoma, loss of SOX10 obviously inhibited cell proliferation, migration, and invasion, as well as the EMT process [30]. In this study, upregulation of SOX10 promoted the migration and invasion of MSCs, and SOX10-overexpressing MSCs were able to more effectively home to the location of the MI and improve heart function. Furthermore, knocking down CXCR4 reversed the promotive effects of SOX10 on the MSC migration and homing, implying that SOX10 promotes MSC migration and homing by upregulating CXCR4.

In summary, SOX10 enhances the migration and homing capacity of MSCs by upregulating the expression of CXCR4, leading to improved outcomes in a rat model of MI. This finding offers a theoretical foundation for developing more effective MSC-based therapies for MI treatment.

## Data Availability

The data generated or analyzed during the study are included in this article, and datasets are available from the corresponding author upon reasonable request.

## Supporting Information

Supporting Information 1 Figure S3: Original images of Western blotting.

Supporting Information 2 Figure S1: Establishment of the MI model before MSC transplantation. a. Echocardiography of cardiac function in a rat MI model. b. Quantification of LVEDD. c. Quantification of LVESD. d. Quantification of LVEF. e. Quantification of LVFS. NS, not significant.

Supporting Information 3 Figure S2: SOX10-overexpressing MSC model and MI model establishment prior to MSC transplantation. -b. RT-qPCR and Western blot detection of SOX10 expression in MSCs transfected with SOX10 or negative control lentivirus. c. Echocardiography to assess cardiac function in MI rats. d. Quantification of LVEDD. e. Quantification of LVESD. f. Quantification of LVEF. g. Quantification of LVFS. NS, not significant.

Supporting Information 4 Raw data for Figure 3b–e, Figure 6b–e, Supporting Information 2: Figure S1b–e, and Supporting Information 3: S2d–g.
