# Supplementary figures and images for: Transcription Factor SOX10 Improves Migration and Homing of MSCs After Myocardial Infarction by Upregulating CXCR4
Source: Stem Cells Int. 2025 May 26;2025:1880402. doi: 10.1155/sci/1880402 (PMC12129602; doi:10.1155/sci/1880402)

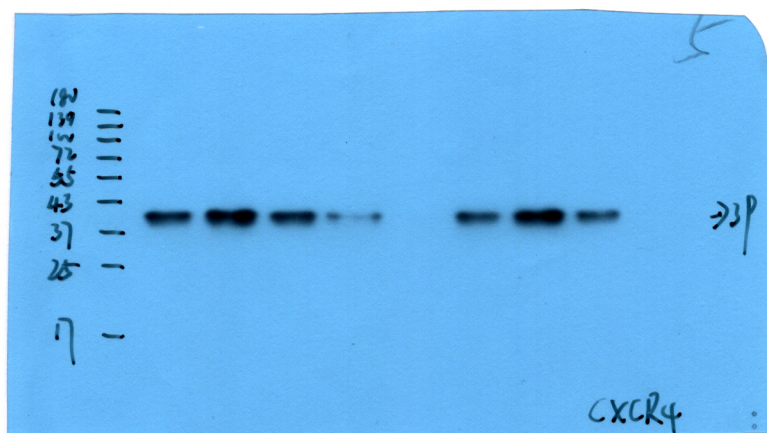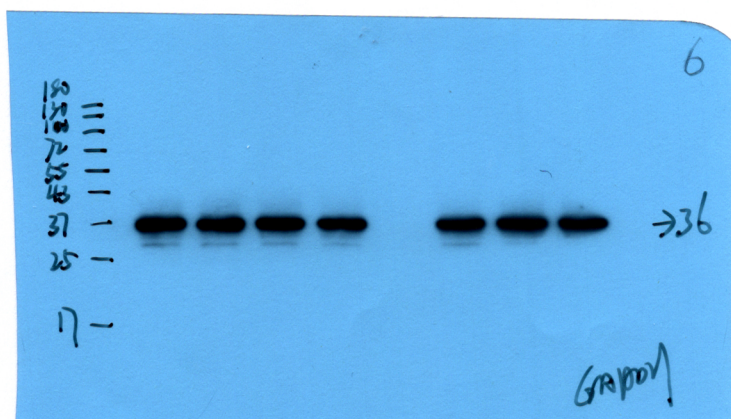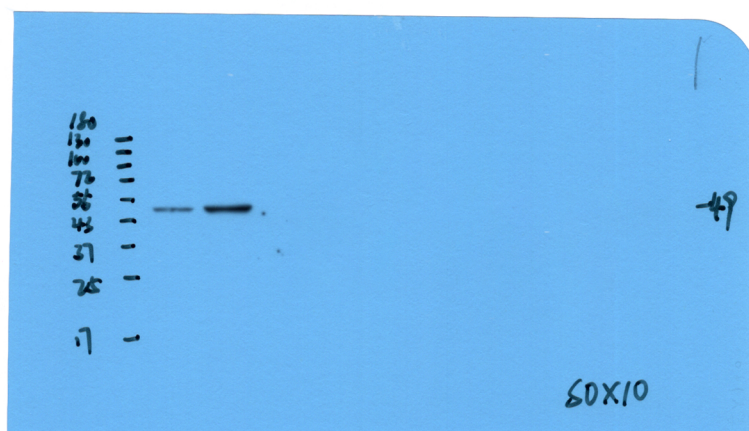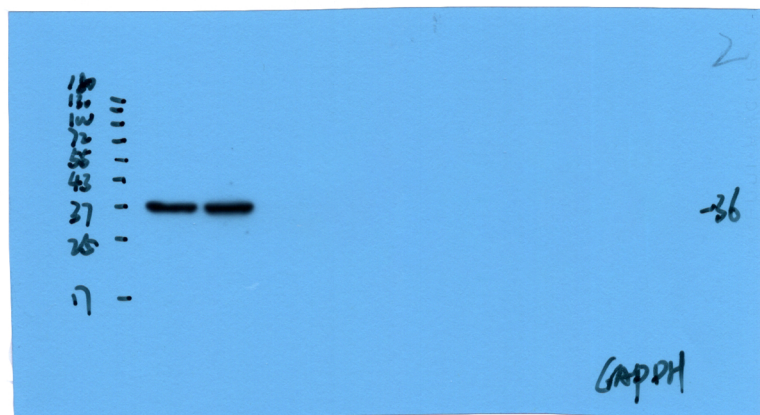

Supplement: Supporting Information 1 — Figure S3: Original images of Western blotting. [file 1880402.f1.pdf]

**A**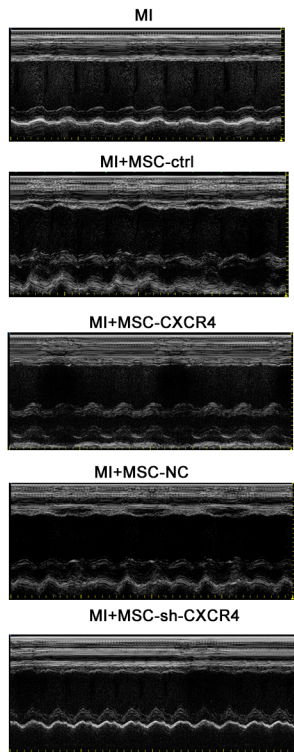**B**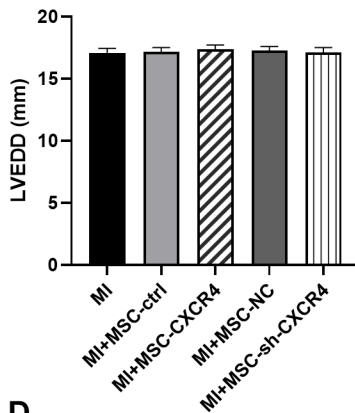**C**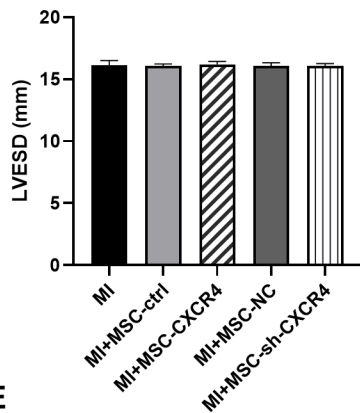**D**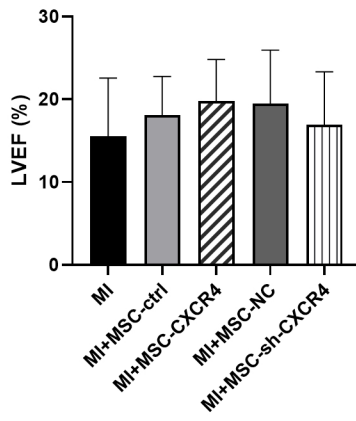**E**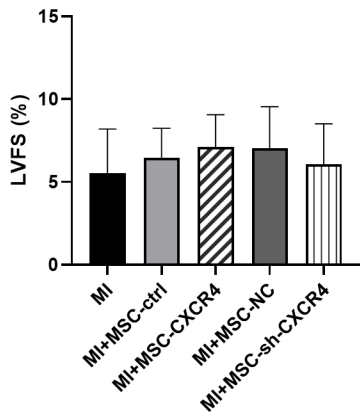

Supplement: Supporting Information 2 — Figure S1: Establishment of the MI model before MSC transplantation. a. Echocardiography of cardiac function in a rat MI model. b. Quantification of LVEDD. c. Quantification of LVESD. d. Quantification of LVEF. e. Quantification of LVFS. NS, not significant. [file 1880402.f2.pdf]

**A**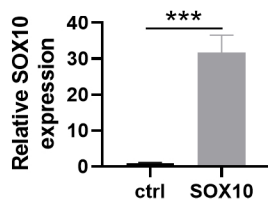**D**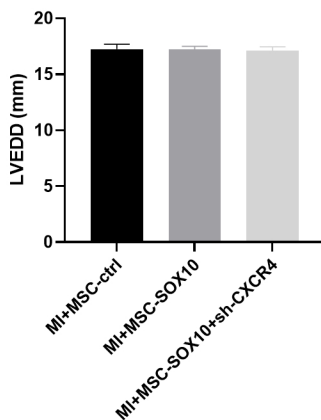**E**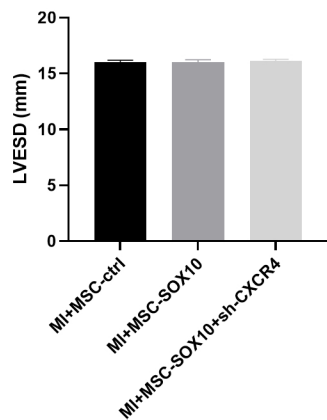**B**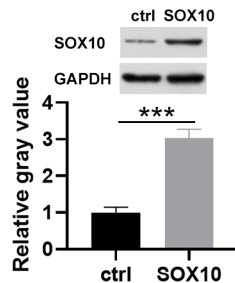**C**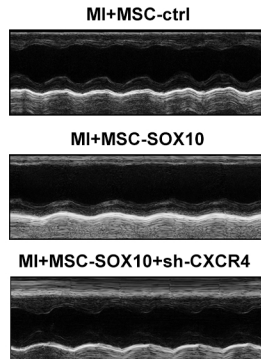**F**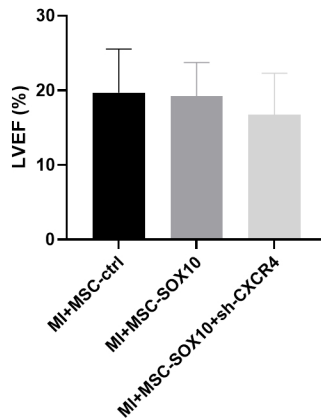**G**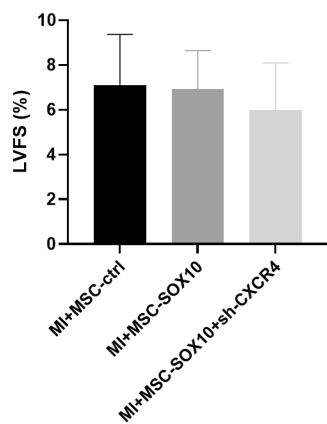

Supplement: Supporting Information 3 — Figure S2: SOX10-overexpressing MSC model and MI model establishment prior to MSC transplantation. -b. RT-qPCR and Western blot detection of SOX10 expression in MSCs transfected with SOX10 or negative control lentivirus. c. Echocardiography to assess cardiac function in MI rats. d. Quantification of LVEDD. e. Quantification of LVESD. f. Quantification of LVEF. g. Quantification of LVFS. NS, not significant. [file 1880402.f3.pdf]
