# Supplementary material for: Transcription Factor SOX10 Improves Migration and Homing of MSCs After Myocardial Infarction by Upregulating CXCR4
Source: Stem Cells Int. 2025 May 26;2025:1880402. doi: 10.1155/sci/1880402 (PMC12129602; doi:10.1155/sci/1880402)
Supplement: Supporting Information 4 — Raw data for Figure 3b–e, Figure 6b–e, Supporting Information 2: Figure S1b–e, and Supporting Information 3: S2d–g. [file 1880402.f4.docx]

The raw data of Fig. 3b-e

| Groups | rat numbers | Times | LVEDD(mm) | LVESE(mm) | LVEF(%) | LVFS(%) |
| --- | --- | --- | --- | --- | --- | --- |
| Sham | 16 | 1 | 7.39 | 4.18 | 81.90 | 43.44 |
|  |  | 2 | 7.61 | 4.17 | 83.55 | 45.20 |
|  |  | 3 | 7.7 | 3.22 | 92.69 | 58.18 |
|  | 4 | 1 | 7.71 | 3.41 | 91.35 | 55.77 |
|  |  | 2 | 6.99 | 3.6 | 86.34 | 48.50 |
|  |  | 3 | 7.86 | 3.72 | 89.40 | 52.67 |
|  | 1 | 1 | 8.51 | 4.38 | 86.37 | 48.53 |
|  |  | 2 | 6.97 | 4.11 | 79.50 | 41.03 |
|  |  | 3 | 8.13 | 4.01 | 88.00 | 50.68 |
|  | 28 | 1 | 7.76 | 4.45 | 81.14 | 42.65 |
|  |  | 2 | 7.99 | 4.01 | 87.36 | 49.81 |
|  |  | 3 | 6.92 | 4.11 | 79.05 | 40.61 |
|  | 2 | 1 | 8.57 | 4.25 | 87.80 | 50.41 |
|  |  | 2 | 8.07 | 3.97 | 88.09 | 50.81 |
|  |  | 3 | 8.46 | 4.18 | 87.94 | 50.59 |
| MI | 17 | 1 | 17.53 | 16.56 | 15.70 | 5.53 |
|  |  | 2 | 16.85 | 16.1 | 12.77 | 4.45 |
|  |  | 3 | 17.11 | 16.43 | 11.46 | 3.97 |
|  | 23 | 1 | 16.69 | 16.44 | 4.43 | 1.50 |
|  |  | 2 | 16.64 | 15.73 | 15.53 | 5.47 |
|  |  | 3 | 16.56 | 16.07 | 8.62 | 2.96 |
|  | 6 | 1 | 17.54 | 15.96 | 24.66 | 9.01 |
|  |  | 2 | 16.58 | 16.11 | 8.27 | 2.83 |
|  |  | 3 | 17.03 | 16.45 | 9.87 | 3.41 |
|  | 5 | 1 | 17.52 | 16.14 | 21.82 | 7.88 |
|  |  | 2 | 17.19 | 15.83 | 21.91 | 7.91 |
|  |  | 3 | 17.56 | 15.68 | 28.80 | 10.71 |
|  | 22 | 1 | 17.24 | 16.44 | 13.29 | 4.64 |
|  |  | 2 | 16.5 | 15.1 | 23.36 | 8.48 |
|  |  | 3 | 17.23 | 16.51 | 12.02 | 4.18 |
| MI+MSC-ctrl | 9 | 1 | 12.84 | 9.05 | 64.99 | 29.52 |
|  |  | 2 | 13.12 | 9.57 | 61.19 | 27.06 |
|  |  | 3 | 12.75 | 9.16 | 62.92 | 28.16 |
|  | 30 | 1 | 13.6 | 9.21 | 68.94 | 32.28 |
|  |  | 2 | 12.51 | 9.52 | 55.93 | 23.90 |
|  |  | 3 | 12.62 | 9.31 | 59.85 | 26.23 |
|  | 10 | 1 | 13.48 | 9.29 | 67.27 | 31.08 |
|  |  | 2 | 13.14 | 9.13 | 66.46 | 30.52 |
|  |  | 3 | 13.22 | 9.27 | 65.52 | 29.88 |
|  | 29 | 1 | 12.4 | 9.12 | 60.21 | 26.45 |
|  |  | 2 | 12.85 | 8.96 | 66.10 | 30.27 |
|  |  | 3 | 12.28 | 9.48 | 53.99 | 22.80 |
|  | 24 | 1 | 13.27 | 9.15 | 67.22 | 31.05 |
|  |  | 2 | 13.38 | 8.92 | 70.37 | 33.33 |
|  |  | 3 | 12.74 | 9.45 | 59.19 | 25.82 |
| MI+MSC-CXCR4 | 27 | 1 | 10.29 | 6.1 | 79.17 | 40.72 |
|  |  | 2 | 9.85 | 6.49 | 71.40 | 34.11 |
|  |  | 3 | 10.06 | 6.22 | 76.36 | 38.17 |
|  | 21 | 1 | 9.55 | 5.57 | 80.16 | 41.68 |
|  |  | 2 | 10.41 | 6.15 | 79.38 | 40.92 |
|  |  | 3 | 10.5 | 5.84 | 82.79 | 44.38 |
|  | 14 | 1 | 9.26 | 5.63 | 77.53 | 39.20 |
|  |  | 2 | 9.36 | 5.65 | 78.01 | 39.64 |
|  |  | 3 | 9.67 | 5.71 | 79.41 | 40.95 |
|  | 20 | 1 | 9.74 | 6.5 | 70.28 | 33.26 |
|  |  | 2 | 9.3 | 6.26 | 69.50 | 32.69 |
|  |  | 3 | 10.46 | 6.17 | 79.48 | 41.01 |
|  | 13 | 1 | 9.11 | 6.51 | 63.51 | 28.54 |
|  |  | 2 | 9.12 | 6.42 | 65.12 | 29.61 |
|  |  | 3 | 9.31 | 6.37 | 67.97 | 31.58 |
| MI+MSC-NC | 3 | 1 | 12.58 | 9.57 | 55.98 | 23.93 |
|  |  | 2 | 13.2 | 9.52 | 62.49 | 27.88 |
|  |  | 3 | 12.49 | 9.36 | 57.91 | 25.06 |
|  | 15 | 1 | 13.53 | 8.98 | 70.76 | 33.63 |
|  |  | 2 | 12.86 | 9.23 | 63.03 | 28.23 |
|  |  | 3 | 13.58 | 9.49 | 65.87 | 30.12 |
|  | 7 | 1 | 12.49 | 9.18 | 60.30 | 26.50 |
|  |  | 2 | 13.15 | 9.23 | 65.42 | 29.81 |
|  |  | 3 | 12.43 | 9.01 | 61.91 | 27.51 |
|  | 11 | 1 | 12.01 | 9.37 | 52.51 | 21.98 |
|  |  | 2 | 13.52 | 9.23 | 68.18 | 31.73 |
|  |  | 3 | 12.29 | 9.15 | 58.73 | 25.55 |
|  | 8 | 1 | 12.2 | 9.23 | 56.70 | 24.34 |
|  |  | 2 | 12.37 | 9.57 | 53.70 | 22.64 |
|  |  | 3 | 12.61 | 9.53 | 56.83 | 24.43 |
| MI+MSC-sh-CXCR4 | 18 | 1 | 14.85 | 12.31 | 43.04 | 17.10 |
|  |  | 2 | 15.4 | 13.23 | 36.60 | 14.09 |
|  |  | 3 | 15.48 | 12.79 | 43.60 | 17.38 |
|  | 13 | 1 | 14.05 | 12.71 | 25.97 | 9.54 |
|  |  | 2 | 14.46 | 12.65 | 33.05 | 12.52 |
|  |  | 3 | 15.04 | 13.58 | 26.39 | 9.71 |
|  | 7 | 1 | 15.11 | 12.82 | 38.92 | 15.16 |
|  |  | 2 | 14.54 | 12.84 | 31.13 | 11.69 |
|  |  | 3 | 14.71 | 12.3 | 41.54 | 16.38 |
|  | 24 | 1 | 15.59 | 13.54 | 34.49 | 13.15 |
|  |  | 2 | 14.37 | 12.42 | 35.44 | 13.57 |
|  |  | 3 | 14.58 | 13.02 | 28.79 | 10.70 |
|  | 17 | 1 | 15.56 | 12.02 | 53.90 | 22.75 |
|  |  | 2 | 14.89 | 12.71 | 37.81 | 14.64 |
|  |  | 3 | 15.48 | 13.52 | 33.38 | 12.66 |

The raw data of Fig. 6b-e

| Groups | rat numbers | Times | LVEDD(mm) | LVESE(mm) | LVEF(%) | LVFS(%) |
| --- | --- | --- | --- | --- | --- | --- |
| MI+MSC-ctrl | 10 | 1 | 12.27 | 8.99 | 60.67 | 26.73 |
|  |  | 2 | 12.97 | 9.79 | 56.99 | 24.52 |
|  |  | 3 | 13.35 | 8.91 | 70.27 | 33.26 |
|  | 1 | 1 | 12.46 | 9.05 | 61.68 | 27.37 |
|  |  | 2 | 12.73 | 9.5 | 58.44 | 25.37 |
|  |  | 3 | 12.08 | 9.86 | 45.62 | 18.38 |
|  | 14 | 1 | 12.16 | 8.93 | 60.39 | 26.56 |
|  |  | 2 | 13.38 | 9.65 | 62.48 | 27.88 |
|  |  | 3 | 13.07 | 8.89 | 68.53 | 31.98 |
|  | 7 | 1 | 12.51 | 9.9 | 50.44 | 20.86 |
|  |  | 2 | 12.67 | 9.32 | 60.20 | 26.44 |
|  |  | 3 | 12.37 | 9.81 | 50.12 | 20.70 |
|  | 4 | 1 | 12.26 | 9 | 60.44 | 26.59 |
|  |  | 2 | 12.9 | 9.9 | 54.80 | 23.26 |
|  |  | 3 | 13.12 | 9.66 | 60.09 | 26.37 |
| MI+MSC-CXCR4 | 12 | 1 | 10.68 | 5.94 | 82.80 | 44.38 |
|  |  | 2 | 9.89 | 6.49 | 71.74 | 34.38 |
|  |  | 3 | 10.47 | 6.28 | 78.42 | 40.02 |
|  | 8 | 1 | 10.22 | 6.59 | 73.19 | 35.52 |
|  |  | 2 | 9.79 | 6.15 | 75.21 | 37.18 |
|  |  | 3 | 10.9 | 6.44 | 79.38 | 40.92 |
|  | 5 | 1 | 10 | 6.76 | 69.11 | 32.40 |
|  |  | 2 | 10.5 | 6.06 | 80.78 | 42.29 |
|  |  | 3 | 10.05 | 6.04 | 78.29 | 39.90 |
|  | 9 | 1 | 10.53 | 6.37 | 77.86 | 39.51 |
|  |  | 2 | 10.27 | 6.69 | 72.36 | 34.86 |
|  |  | 3 | 10.81 | 6.17 | 81.41 | 42.92 |
|  | 2 | 1 | 10.8 | 6.48 | 78.40 | 40.00 |
|  |  | 2 | 10.74 | 6.3 | 79.82 | 41.34 |
|  |  | 3 | 10.56 | 6.04 | 81.29 | 42.80 |
| MI+MSC-SOX10+sh-CXCR4 | 13 | 1 | 12.74 | 9.6 | 57.21 | 24.65 |
|  |  | 2 | 13.26 | 10.34 | 52.58 | 22.02 |
|  |  | 3 | 12.86 | 10.17 | 50.54 | 20.92 |
|  | 6 | 1 | 12.3 | 9.45 | 54.65 | 23.17 |
|  |  | 2 | 12.21 | 9.96 | 45.72 | 18.43 |
|  |  | 3 | 12.18 | 9.63 | 50.58 | 20.94 |
|  | 3 | 1 | 12.52 | 9.39 | 57.81 | 25.00 |
|  |  | 2 | 12.33 | 10.2 | 43.39 | 17.27 |
|  |  | 3 | 12.97 | 10.04 | 53.61 | 22.59 |
|  | 11 | 1 | 13.05 | 8.91 | 68.17 | 31.72 |
|  |  | 2 | 12.2 | 10.18 | 41.90 | 16.56 |
|  |  | 3 | 12.38 | 10.12 | 45.38 | 18.26 |
|  | 15 | 1 | 11.82 | 9.38 | 50.02 | 20.64 |
|  |  | 2 | 13.18 | 9.23 | 65.66 | 29.97 |
|  |  | 3 | 12.44 | 9.52 | 55.18 | 23.47 |

The raw data of Supplementary Fig. 1b-e

| Groups | rat numbers | Times | LVEDD(mm) | LVESE(mm) | LVEF(%) | LVFS(%) |
| --- | --- | --- | --- | --- | --- | --- |
| MI | 17 | 1 | 17.53 | 16.56 | 15.70 | 5.53 |
|  |  | 2 | 16.85 | 16.1 | 12.77 | 4.45 |
|  |  | 3 | 17.11 | 16.43 | 11.46 | 3.97 |
|  | 23 | 1 | 16.69 | 16.44 | 4.43 | 1.50 |
|  |  | 2 | 16.64 | 15.73 | 15.53 | 5.47 |
|  |  | 3 | 16.56 | 16.07 | 8.62 | 2.96 |
|  | 6 | 1 | 17.54 | 15.96 | 24.66 | 9.01 |
|  |  | 2 | 16.58 | 16.11 | 8.27 | 2.83 |
|  |  | 3 | 17.03 | 16.45 | 9.87 | 3.41 |
|  | 5 | 1 | 17.52 | 16.14 | 21.82 | 7.88 |
|  |  | 2 | 17.19 | 15.83 | 21.91 | 7.91 |
|  |  | 3 | 17.56 | 15.68 | 28.80 | 10.71 |
|  | 22 | 1 | 17.24 | 16.44 | 13.29 | 4.64 |
|  |  | 2 | 16.5 | 15.1 | 23.36 | 8.48 |
|  |  | 3 | 17.23 | 16.51 | 12.02 | 4.18 |
| MI+MSC-ctrl | 9 | 1 | 17.58 | 16.07 | 23.62 | 8.59 |
|  |  | 2 | 16.75 | 16.01 | 12.68 | 4.42 |
|  |  | 3 | 17.39 | 15.85 | 24.28 | 8.86 |
|  | 30 | 1 | 17.79 | 16.22 | 24.21 | 8.83 |
|  |  | 2 | 17.08 | 15.81 | 20.69 | 7.44 |
|  |  | 3 | 17.11 | 16.01 | 18.07 | 6.43 |
|  | 10 | 1 | 16.98 | 16.07 | 15.23 | 5.36 |
|  |  | 2 | 16.86 | 16.26 | 10.30 | 3.56 |
|  |  | 3 | 16.9 | 16.23 | 11.43 | 3.96 |
|  | 29 | 1 | 17.18 | 15.87 | 21.18 | 7.63 |
|  |  | 2 | 16.99 | 16.07 | 15.38 | 5.41 |
|  |  | 3 | 16.9 | 15.91 | 16.56 | 5.86 |
|  | 24 | 1 | 16.91 | 15.94 | 16.24 | 5.74 |
|  |  | 2 | 17.79 | 16.27 | 23.50 | 8.54 |
|  |  | 3 | 17.36 | 16.26 | 17.83 | 6.34 |
| MI+MSC-CXCR4 | 27 | 1 | 17.77 | 16.23 | 23.81 | 8.67 |
|  |  | 2 | 17.67 | 16.02 | 25.48 | 9.34 |
|  |  | 3 | 17.5 | 15.8 | 26.40 | 9.71 |
|  | 21 | 1 | 17.51 | 16.24 | 20.22 | 7.25 |
|  |  | 2 | 17.52 | 16.27 | 19.91 | 7.13 |
|  |  | 3 | 17.24 | 16.16 | 17.64 | 6.26 |
|  | 14 | 1 | 16.95 | 16.06 | 14.94 | 5.25 |
|  |  | 2 | 17.8 | 16.35 | 22.50 | 8.15 |
|  |  | 3 | 16.74 | 16.08 | 11.37 | 3.94 |
|  | 20 | 1 | 16.94 | 16.28 | 11.24 | 3.90 |
|  |  | 2 | 17.14 | 16.39 | 12.56 | 4.38 |
|  |  | 3 | 17.19 | 15.92 | 20.57 | 7.39 |
|  | 13 | 1 | 17.61 | 15.99 | 25.14 | 9.20 |
|  |  | 2 | 17.24 | 16.25 | 16.26 | 5.74 |
|  |  | 3 | 17.71 | 16.02 | 25.98 | 9.54 |
| MI+MSC-NC | 3 | 1 | 17.45 | 15.81 | 25.63 | 9.40 |
|  |  | 2 | 17.17 | 15.82 | 21.78 | 7.86 |
|  |  | 3 | 17.6 | 16.32 | 20.27 | 7.27 |
|  | 15 | 1 | 16.64 | 16.06 | 10.10 | 3.49 |
|  |  | 2 | 17 | 15.99 | 16.79 | 5.94 |
|  |  | 3 | 17.42 | 16.17 | 20.02 | 7.18 |
|  | 7 | 1 | 17.53 | 15.99 | 24.11 | 8.78 |
|  |  | 2 | 17.4 | 16.26 | 18.40 | 6.55 |
|  |  | 3 | 16.9 | 16.15 | 12.73 | 4.44 |
|  | 11 | 1 | 17.23 | 16.19 | 17.04 | 6.04 |
|  |  | 2 | 16.93 | 16.17 | 12.87 | 4.49 |
|  |  | 3 | 17.75 | 16.06 | 25.93 | 9.52 |
|  | 8 | 1 | 17.05 | 16.4 | 11.01 | 3.81 |
|  |  | 2 | 17.5 | 16.25 | 19.93 | 7.14 |
|  |  | 3 | 17.56 | 15.98 | 24.64 | 9.00 |
| MI+MSC-sh-CXCR4 | 18 | 1 | 17.23 | 16.18 | 17.19 | 6.09 |
|  |  | 2 | 17.53 | 16.22 | 20.79 | 7.47 |
|  |  | 3 | 16.55 | 15.93 | 10.82 | 3.75 |
|  | 13 | 1 | 16.8 | 16.12 | 11.66 | 4.05 |
|  |  | 2 | 17.11 | 16.4 | 11.94 | 4.15 |
|  |  | 3 | 16.94 | 16.24 | 11.89 | 4.13 |
|  | 7 | 1 | 17.41 | 16.13 | 20.47 | 7.35 |
|  |  | 2 | 17.7 | 16.26 | 22.47 | 8.14 |
|  |  | 3 | 16.99 | 16.22 | 12.99 | 4.53 |
|  | 24 | 1 | 16.63 | 15.96 | 11.61 | 4.03 |
|  |  | 2 | 16.83 | 16.23 | 10.32 | 3.57 |
|  |  | 3 | 17.74 | 15.97 | 27.05 | 9.98 |
|  | 17 | 1 | 16.62 | 16.02 | 10.44 | 3.61 |
|  |  | 2 | 17.47 | 15.97 | 23.61 | 8.59 |
|  |  | 3 | 17.08 | 16.11 | 16.09 | 5.68 |

The raw data of Supplementary Fig. 2d-g

| Groups | rat numbers | Times | LVEDD(mm) | LVESE(mm) | LVEF(%) | LVFS(%) |
| --- | --- | --- | --- | --- | --- | --- |
| MI+MSC-ctrl | 10 | 1 | 17.65 | 16.01 | 25.37 | 9.29 |
|  |  | 2 | 17 | 16.04 | 16.00 | 5.65 |
|  |  | 3 | 17.56 | 16.39 | 18.69 | 6.66 |
|  | 1 | 1 | 16.91 | 15.92 | 16.56 | 5.85 |
|  |  | 2 | 16.97 | 16.21 | 12.84 | 4.48 |
|  |  | 3 | 17.23 | 15.88 | 21.71 | 7.84 |
|  | 14 | 1 | 16.91 | 16.19 | 12.24 | 4.26 |
|  |  | 2 | 16.92 | 16.05 | 14.65 | 5.14 |
|  |  | 3 | 17.72 | 16.13 | 24.58 | 8.97 |
|  | 7 | 1 | 17.38 | 15.85 | 24.15 | 8.80 |
|  |  | 2 | 17.8 | 15.83 | 29.66 | 11.07 |
|  |  | 3 | 16.68 | 15.87 | 13.87 | 4.86 |
|  | 4 | 1 | 17.76 | 16.07 | 25.92 | 9.52 |
|  |  | 2 | 16.67 | 15.87 | 13.72 | 4.80 |
|  |  | 3 | 17.8 | 16.12 | 25.73 | 9.44 |
| MI+MSC-CXCR4 | 12 | 1 | 17.31 | 15.81 | 23.81 | 8.67 |
|  |  | 2 | 16.93 | 15.92 | 16.85 | 5.97 |
|  |  | 3 | 16.92 | 15.83 | 18.11 | 6.44 |
|  | 8 | 1 | 16.9 | 16.13 | 13.06 | 4.56 |
|  |  | 2 | 17.61 | 16.31 | 20.55 | 7.38 |
|  |  | 3 | 17.3 | 15.95 | 21.63 | 7.80 |
|  | 5 | 1 | 17.1 | 15.84 | 20.52 | 7.37 |
|  |  | 2 | 16.94 | 16.04 | 15.11 | 5.31 |
|  |  | 3 | 17.75 | 15.8 | 29.47 | 10.99 |
|  | 9 | 1 | 16.93 | 15.8 | 18.72 | 6.67 |
|  |  | 2 | 17.37 | 16.28 | 17.67 | 6.28 |
|  |  | 3 | 17.41 | 16.29 | 18.08 | 6.43 |
|  | 2 | 1 | 17.43 | 16.39 | 16.85 | 5.97 |
|  |  | 2 | 17.47 | 15.85 | 25.32 | 9.27 |
|  |  | 3 | 16.94 | 16.15 | 13.35 | 4.66 |
| MI+MSC-SOX10+sh-CXCR4 | 13 | 1 | 16.91 | 16.08 | 14.01 | 4.91 |
|  |  | 2 | 17.04 | 16.35 | 11.66 | 4.05 |
|  |  | 3 | 17.19 | 16.14 | 17.23 | 6.11 |
|  | 6 | 1 | 17.46 | 15.88 | 24.77 | 9.05 |
|  |  | 2 | 16.54 | 15.96 | 10.16 | 3.51 |
|  |  | 3 | 17.09 | 16.4 | 11.63 | 4.04 |
|  | 3 | 1 | 16.74 | 16.03 | 12.19 | 4.24 |
|  |  | 2 | 17.17 | 16.11 | 17.40 | 6.17 |
|  |  | 3 | 16.9 | 16.16 | 12.57 | 4.38 |
|  | 11 | 1 | 17.75 | 16.03 | 26.34 | 9.69 |
|  |  | 2 | 17.11 | 15.86 | 20.35 | 7.31 |
|  |  | 3 | 17.23 | 16.31 | 15.18 | 5.34 |
|  | 15 | 1 | 16.84 | 16.18 | 11.30 | 3.92 |
|  |  | 2 | 17.69 | 16.14 | 24.05 | 8.76 |
|  |  | 3 | 17.29 | 15.87 | 22.67 | 8.21 |
